# The UTHealth Houston Adult Cardiovascular Genomics Certificate Program: Efficacy and Impact on Healthcare Professionals

**DOI:** 10.21203/rs.3.rs-4469272/v1

**Published:** 2024-06-13

**Authors:** Melyssa Garner, Bansari Rajani, Priyanka Vaidya, Samer Abu Dayeh, Alana C. Cecchi, Christina C. Miyake, Vicki Huff, Matthew Wanat, Elisabeth Wang, Leonie M. Kurzlechner, Andrew P. Landstrom, Daniel An, Yafen Liang, Mousumi Moulik, Timothy C. Wong, Shane R. Cunha, Ashley Cannon, R. Lynn Holt, Dianna M. Milewicz, Siddharth K. Prakash

**Affiliations:** University of Alabama at Birmingham; University of Texas Health Science Center at Houston; University of Texas Health Science Center at Houston; University of Texas Health Science Center at Houston; University of Texas Health Science Center at Houston; Baylor College of Medicine; University of Texas MD Anderson Cancer Center; University of Houston College of Pharmacy; University of Houston College of Pharmacy; Duke University School of Medicine; Duke University School of Medicine; University of Texas Health Science Center at Houston; University of Texas Health Science Center at Houston; University of Pittsburgh School of Medicine; University of Pittsburgh School of Medicine; University of Texas Health Science Center at Houston; University of Alabama at Birmingham; University of Alabama at Birmingham; University of Texas Health Science Center at Houston; University of Texas Health Science Center at Houston

**Keywords:** Genomic medicine, Professional development, Continuing medical education, Cardiovascular genetics

## Abstract

**Background:**

The demand for genetic services has outpaced the availability of resources, challenging clinicians untrained in genetic integration into clinical decision-making. The UTHealth Adult Cardiovascular Genomics Certificate (CGC) program trains non-genetic healthcare professionals to recognize, assess, and refer patients with heritable cardiovascular diseases. This asynchronous online course includes 24 modules in three tiers of increasing complexity, using realistic clinical scenarios, interactive dialogues, quizzes, and tests to reinforce learning. We hypothesized that the CGC will increase genomic competencies in this underserved audience and encourage applying genomic concepts in clinical practice.

**Methods:**

Required course evaluations include pre- and post-assessments, knowledge checks in each module, and surveys for module-specific feedback. After 6 months, longitudinal feedback surveys gathered data on the long-term impact of the course on clinical practice and conducted focused interviews with learners.

**Results:**

The CGC was accredited in September 2022. Principal learners were nurses (24%), nurse practitioners (21%), physicians (16%), and physician assistants. Scores of 283 learners in paired pre- and post-assessments increased specific skills related to recognizing heritable diseases, understanding inheritance patterns, and interpreting genetic tests. Interviews highlighted the CGC’s modular structure and linked resources as key strengths. Learners endorsed confidence to use genetic information in clinical practice, such as discussing genetic concepts and risks with patients and referring patients for genetic testing. Learners were highly likely to recommend the CGC to colleagues, citing its role in enhancing heritable disease awareness.

**Conclusions:**

The CGC program effectively empowers non-genetic clinicians to master genomic competencies, fostering collaboration to prevent deaths from heritable cardiovascular diseases, and potentially transforming healthcare education and clinical practice.

## Introduction

The demand for genetic services has increased exponentially since the completion of the Human Genome Project. The workforce population of clinical geneticists and genetic counselors has failed to sufficiently meet this demand. Approximately two clinical geneticists provide care per one million patients in the United States (Maiese et al., 2019). By 2025, there will be less than one certified genetic counselor per 100,000 patients seeking genetic services (Hoskovec et al., 2018). Due to the workforce shortage of genetics providers, genetic tests are increasingly ordered by healthcare providers who lack formal genetics training (Shields et al., 2008; Truong et al., 2021). These providers have become gatekeepers of genetic services and partners in providing care for patients with genetic conditions.

Insufficient knowledge of genetic concepts by providers may limit the use of genetic information in clinical practice. Moreover, providers are generally unprepared to counsel patients (Klitzman et al., 2013; Prochniak et al., 2012). Gaps in provider knowledge have been shown to result in adverse medical, psychological, and financial outcomes for patients and families (Bensend et al., 2014; Brierley et al., 2012; Farmer et al., 2019; Vadaparampil et al., 2015). Non-genetic healthcare providers have expressed needs for additional education to supplement these knowledge gaps (Carroll et al., 2016; Cusack et al., 2021; Diamonstein et al., 2018; Houwink et al., 2011).

Continuing education in genomic medicine frequently incorporates experiential, hands-on learning that emphasizes relevant skills for clinical practice. Educational models developed to address gaps in genetics knowledge are primarily focused on oncology (Blazer et al., 2012; Carroll et al., 2011; Houwink et al., 2014) or pediatrics (Al-Jasmi et al., 2010; Reed et al., 2016), where the role of genetics is more established. Although the utility of these models is well documented, relatively few resources are available to practitioners in newer and more rapidly expanding fields, such as cardiogenomics. The field of cardiogenomics was made possible by the identification of pathogenic variants in genes that cause heritable cardiovascular diseases, such as thoracic aortic aneurysms and dissections (TAD), hyperlipidemias, and cardiomyopathies. Identification of causative genes for TAD led to personalized surveillance and therapies to prevent deaths from acute vascular dissections (Isselbacher et al., 2022; Milewicz et al., 2021). Use of this genetic data improves outcomes and may save lives by identifying individuals at risk before they develop life-threatening cardiovascular complications. Now that commercial genetic tests have entered the clinical mainstream, non-genetics providers are increasingly confronted with clinically meaningful and potentially lifesaving genetic information.

Primary care providers frequently make the initial contact with adult patients who have heritable cardiovascular disease, and their limited cardiogenomics training may delay diagnosis and provision of guideline-recommended care to this population. Failure to recognize these patients may result in increased risk of adverse cardiovascular events or death. Delayed recognition of inherited conditions is commonly fueled by improper documentation of family history (Christianson et al., 2012; Mehrabi et al., 2016; Taber et al., 2020; Zazove et al., 2015) or difficulty assessing and communicating risk (Flynn et al., 2010; Vadaparampil et al., 2015). Continuing education programs in adult cardiovascular genetics are needed to supplement the gaps in provider knowledge with the goal to improve identification and management of patients with a genetic cardiovascular disease.

The UTHealth Houston Cardiovascular Genomics Certificate Program (CGC) was developed to address these gaps in provider knowledge and fulfill an unmet need for adult cardiovascular genetics education. The course consists of free online educational models about the genetic basis of heritable cardiovascular disease. The target audience is adult care providers without genetics expertise, including physicians, residents, nurses, and advanced practice providers. The CGC is intended to decrease disparities in access to genetics education, increase awareness of inherited cardiovascular diseases, and improve knowledge of genetic counseling and genomic medicine to enable more adult patients to receive timely diagnosis and appropriate care.

## Methods

### Program Development

The primary objectives of the CGC are closely aligned with the NHGRI GenomeEd competencies for healthcare providers (Korf et al., 2014)(Table 1). The overall goals of the program are to fulfill an unmet need for adult cardiovascular genetics, to increase awareness of heritable cardiovascular disease by all health professionals, to improve the recognition and referral of patients with heritable cardiovascular diseases, and to innovate genomics education by featuring case-based modules that address relevant issues about the delivery of personalized genomic medicine in daily clinical cardiovascular practice.

The CGC was founded by collaborative stakeholders at Baylor College of Medicine, the University of Houston College of Pharmacy, and UTHealth Houston, including McGovern Medical School, the Graduate School of Biomedical Sciences, the School of Public Health, the School of Biomedical Informatics, the Masters Genetic Counseling program, and the Cizik School of Nursing. Program faculty include experts in the fields of molecular and human genetics, cardiology, congenital and inherited cardiac disease, and genetic counseling. Course development was funded by an administrative supplement to the UTHealth Houston Clinical and Translational Science Award from the National Center for Advancing Translational Sciences and the National Human Genome Research Institute (NOT-HG-20–020) specifically intended for the implementation of training modules in genomic medicine for healthcare professionals.

CGC modules were developed in partnership with the Office of Educational Programs at McGovern Medical School, a part of UTHealth Houston, using the Canvas Catalog platform. Canvas is an online learning management system widely used by educators. The Canvas interface allows for development and delivery of online modules that include registration tracking, interactive quizzes, course reviews, and post-course assessments. Anyone who registers on Canvas can access the published modules at https://uthealth.catalog.instructure.com/browse/ms/courses/acgcp. The educational content of CGC modules is partly based on the genomics education toolkits developed by the Inter-Society Coordinating Committee for Practitioner Education in Genomics (ISCC-PEG). Expertise from ISCC-PEG and materials from the Training Residents in Genomics (TRIG) working group on case-based applications of genomic concepts to heritable diseases were implemented in module development (Haspel, 2013).

In the first year of program development, the modules were piloted to target audiences at UTHealth Houston: internal medicine faculty, family medicine faculty in the Community Health program, genetic counselors, cardiothoracic and vascular surgeons, anesthesiologists, and nurses. Participants informally provided feedback about the content and intelligibility of the modules, interactive style preferences, and applicability to clinical practice. In the second year of program development, the modules were debuted in the clinical and continuing education programs at UTHealth Houston, and the course was made available to outside learners. The CGC team and specialty content experts continue to update the modules at regular intervals.

### Data Analysis

Data on page views, page interactions, and quiz scores were extracted from Canvas Catalog and analyzed using descriptive statistics. Data from module surveys, CME/CNE evaluations, and long-term feedback surveys were extracted from Qualtrics and REDCap. Pre- and post-course scores were compared using chi-squared tests or t-tests as appropriate. Focused interviews with learners who completed the course or claimed CME credit were analyzed using Taguette software (Supplemental Data).

## Results

### Course Structure

**Figure 1:**
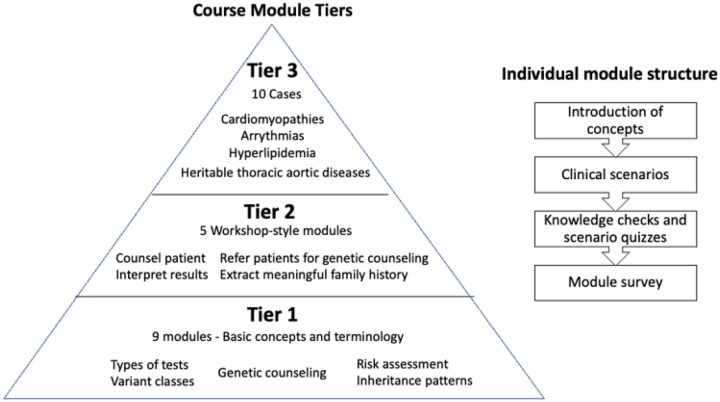
Structure of course module tiers and individual modules A graphic description of the tiers of the course modules and the individual structure of modules. Learners start with basic concepts and terminology in tier one and apply increasing amounts of knowledge as they progress to the clinical cases of tier 3. Each module consists of an introduction of concepts, clinical scenarios, knowledge checks and scenario quizzes, and a module feedback survey.

The CGC consists of 24 modules divided into three tiers of increasing knowledge application across heritable cardiovascular diseases. By the end of the course, learners can use family history and pedigrees to explain heritability, identify patients and families that would benefit from genetic counseling referrals, select appropriate genetic tests for different clinical scenarios, and interpret the clinical significance of genetic variants. To unlock the course modules, participants take a pre-course assessment that establishes their baseline knowledge of genetics and cardiovascular disease. Learners begin with short presentations on basic genetic concepts and terminology (Tier 1), progress to workshop-style activities where they apply these concepts (Tier 2) and conclude the course by completing longer clinical cases that require them to contextualize these concepts in the practice of genomic medicine (Tier 3). Each module consists of an introduction of concepts followed by several clinical scenarios that illustrate the application of these concepts to daily practice. The scenarios are embedded with knowledge checks and quizzes to reinforce learning. Feedback surveys are presented at the end of each module to assess learner confidence and understanding, module difficulty, completion time, and suggestions for improvement. The course modules are built to stand alone but can be combined flexibly according to individual interests (Fig. 1).

The topics of the nine Tier 1 modules include genetic terminology, inheritance, risk assessment, and understanding genetic test reports. Course learners are introduced to the profession of genetic counseling in Tier 1 and apply genetic counseling skills throughout Tier 2 and Tier 3. In the five Tier 2 modules, course learners practice extracting meaningful information from family histories, interpreting genetic test results, identifying and counseling relatives at risk for heritable cardiovascular diseases, and changing medication dosage based on pharmacogenetic test results. Learners also become familiar with indications for genetic counseling referral and the value of consultations with laboratory genetic counselors. Tier 3 cases require learners to synthesize concepts from Tiers 1 and 2 in 10 realistic genetic counseling scenarios involving heritable cardiovascular diseases that are frequently encountered in clinical practice, such as cardiomyopathies, heritable thoracic aortic diseases, arrythmias, and hyperlipidemias.

**Figure 2:**
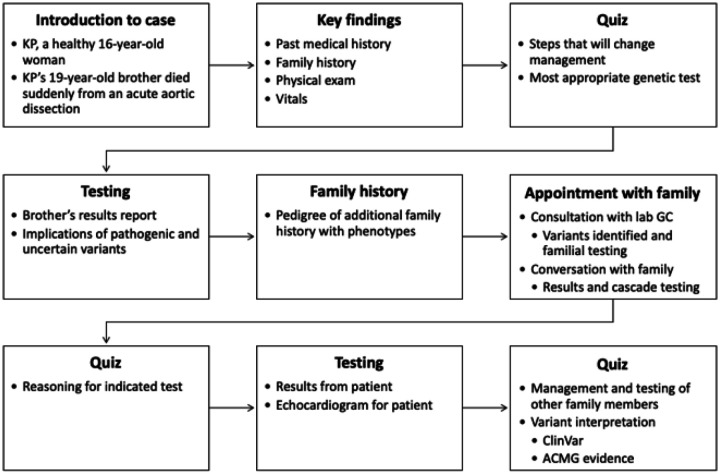
Tier 3 Case 3 Module In Case 3, learners are introduced to the patient, KP, who is an apparently healthy 16-year-old woman with no known medical or cardiovascular conditions. She was referred after her 19-year-old brother died suddenly due to an acute aortic dissection. The key findings of the case are presented, including the medical and family history, physical examination findings, and diagnostic test results. A quiz asks what next step is likely to change management and what genetic test may be most appropriate. The genetic testing results and an expanded pedigree are then presented. Prior to the appointment, the clinician consults with a laboratory genetic counselor who discusses the interpretation of the genetic test results. Learners interactively participate in a counseling session with the patient and her mother that contextualizes the meaning of the test results and the relevance of cascade testing to the family. The final quiz consists of several questions about follow up management, cascade testing, and variant interpretation utilizing data from ClinVar and ACMG guidelines.

In Tier 3 Case 3, an apparently healthy 16-year-old woman with no past medical history was referred to the cardiovascular genetics clinic after her 19-year-old brother died suddenly due to an acute aortic dissection (Fig. 2). After reviewing the family history, exam findings and diagnostic test results, learners decide on an appropriate genetic test. Learners interactively participate in a dialog with the laboratory genetic counsellor, who reviews and interprets the test results, and in a counseling session with the family, which contextualizes the meaning of the results and the relevance of cascade testing. The module test requires learners to assess variant pathogenicity using evidence from clinical databases and to evaluate options for subsequent clinical management.

To claim a Certificate of completion, learners must complete at least three Tier 1 modules, at least two Tier 2 modules and at least three Tier 3 cases. Learners who complete the entire course can claim up to 8.5 credit hours of continuing medical education (CME) or continuing nursing education (CNE) credit. Each individual module is worth credit based on the estimated time of completion of the module. The allotted accreditation time for Tier 1 (5 minutes), Tier 2 (15 minutes) and Tier 3 (30 minutes) modules reflects their increasing complexity. Learners can complete the course at their own pace. A post-course assessment evaluates the impact of the course on genetic knowledge and genomic medicine competencies.

### Impact of CGC

By January 2024, 283 learners had interacted with the CGC, viewing a median of 250 course pages (IQR 13–207), four modules, and 20 quizzes. Learners spent the most time and attempted the most quiz questions (65–99) in Tier 1 modules. Twenty learners claimed CME or CNE credit hours. Mean scores increased by more than 20–70% on the post-course assessment test. Competencies that increased the most were: understanding genetic tests, knowing indications for genetic testing, interpretation of family history and pedigrees, appropriate indications for cascade testing or referrals to genetic counseling, and distinguishing between variant types (Table 2).

We received a mean of 15 feedback survey responses to each module, primarily from nurse practitioners (Table 3). The mean time to complete each module was 19 minutes (14–22). The CGC was rated as highly satisfactory by more than 95% of respondents. Survey participants agreed that the course content was easy to understand and addressed issues that are relevant to their clinical practice. More than 70% of learners reported that the course increased their confidence to practice genomic competencies, such as applying genetic information to make clinical decisions or consulting with genetic professionals. These results were durable at 6 months, when most respondents indicated that they had counselled patients about their genetic risks and had recommended or ordered clinical genetic tests.

## Discussion

The use of genetic information in healthcare is rapidly expanding at a rate that the genetics workforce alone cannot currently meet. Genetic testing is now a Class I guideline recommendation for many diseases, but most practitioners are not comfortable with ordering or interpreting genetic tests as part of routine clinical care (Khoury and Dotson, 2021). In a recent survey of practicing cardiologists, 81% of providers who ordered genetic tests for their patients identified as non-genetic professionals (Jacome et al., 2022). Continuing education is therefore essential for providers to apply new genetic discoveries to clinical practice, particularly for adult providers who frequently lack formal training in genetics. The UTHealth Houston Cardiovascular Genomics Certificate program (CGC) is intended as an innovative educational resource to address these knowledge gaps. The goal of the CGC is for providers to improve recognition, diagnosis, and management of patients with genetic cardiovascular diseases. The program is free and self-paced and provides CME or CNE credit to learners.

The CGC occupies a unique niche in continuing cardiogenetic education for adult providers. Popular websites for the CardioGenomic Testing Alliance (CGTA) and The American College of Cardiology (ACC) feature instructional videos and links to educational resources about genetic testing but do not provide an integrated course experience for learners. Most CGTA and ACC testing scenarios involve specialist pediatric or adult congenital heart disease cases that are rarely encountered by general clinicians. In contrast to the Northwestern/Jackson Laboratory course “Implementing Cardiogenomics in Clinical Practice,” the CGC includes a broader range of clinical scenarios that are more frequently encountered in clinical practice, such as collaboration with genetic professionals, risk assessment, cascade screening of family members, management of patients with variants of uncertain significance, and reclassification of variants over time. Practitioners ranked these topics as highly desirable for an educational program, and limitations in these areas may explain why genetic education has been slow to penetrate the clinical cardiovascular community (Jacome et al, 2022).

Analysis of assessment data shows that learners achieved substantial genomic competencies after taking the CGC, such as interpretation of genetic information, assessment of heritability, and indications for genetic counseling. Correspondingly, learners expressed a high level of confidence that they would be able to adapt genomic competencies to their clinical practice. These observations proved to be durable in follow up surveys. At six months, learners endorsed increased confidence to interpret genetic test results, communicate genetic information to patients, implement genetic information in treatment plans, and identify candidates for genetic testing. These lasting impacts can benefit both providers and patients. Providers will recognize opportunities to refer patients to genetic counseling or to consult with genetic professionals. Patients may benefit from treatment plans that are personalized based on genetic information, and family members who carry pathogenic variants can be identified in time to prevent disease-related complications.

The CGC takes an innovative approach to genetics education with an emphasis on genetic counseling and collaborative assessment of patients. Clinical scenarios in the CGC model interprofessional collaboration with genetic counselors by providing learners with opportunities to refer patients to genetic counseling and to recognize the value of consultations with laboratory genetic counselors. Increased understanding and integration of genomic medicine by non-genetics healthcare providers will help ease the burden on genetic professionals.

Currently the CGC does not include many cardiovascular diseases with actionable genetic information and does not provide content about ethical, legal, or social issues (ELSI). In feedback surveys, learners requested more case scenarios illustrating genomic concepts and an increased emphasis on pharmacogenomics. To address these limitations, the course development team plans to expand course content to include ELSI, enhanced genetic counseling scenarios, additional cardiovascular diseases, and polygenic risk scores while improving the course experience for learners. The long-term plan is to develop a livestreamed course with case presentations that deepen and reinforce genetic concepts introduced in the modules.

## Figures and Tables

**Table 1:** UTHealth Houston Cardiovascular Genomics Certificate Program (CGC) competencies and corresponding GenomeEd Competencies (https://genomicseducation.net/competency/physician). CMA: chromosomal microarray analysis, WES: whole exome sequencing; WGS: whole genome sequencing.

CGC Competencies	NHGRI GenomeEd Competencies
**C1: Genetic Testing** **Describe the types of genetic tests that are available and indications for their use: chromosome analysis, CMA, panels, WES, WGS**	**2A1, 2A6, 4A1** **Use genomic testing appropriately to guide patient management**
**C2: Heritability and History** **Obtain an appropriate family history and interpret a pedigree.**	**1A1–1A6, 1B1–2, 1C1, 1F1–2** **Elicit, document, and act on relevant family history pertinent to the patient’s clinical status**
**C3: Indications and Referrals** Refer a family for cascade genetic testing. Identify indications to refer a patient for genetic counseling.	**1G1, 2G1, 4G1, 2A7, 2A8, 3G2** Interprofessional collaboration, personal and professional development Make appropriate referrals based on genomic screening and testing results
**C4: Test Interpretation**Correctly interpret genetic test reports: Describe classifications of genetic variants Find and interpret information about the clinical significance of genetic variants. Use genetic data to inform clinical decisionmaking about the choice or timing of therapies.	1. **2B1–3,4C1** a. Practice-based learning and improvement 2. **2B5,3A1–3, 3C2** a. Use genomic information to make treatment decisions 3. **2C1, 2C2, 3B3–5, 3D1, 4B3** a. Incorporate genomic results into patient health record and care plan, discuss results-based therapeutic approaches with patient

**Table 2:** Comparative analysis of proficiencies in genomic competencies, with paired pre-course and post-course completion data.

Core Competencies	Concepts	Pre%	Post%	*P*
Understand how genetic variants are Classified Distinguish between types of genetic tests that are available and indications	Interpretation of genotypes	50	81	0.0002
	Copy Number Variants	27	54	0.0008
	Next Generation Sequencing	22	44	0.01
Assess the heritability of disease by obtaining an appropriate family history and interpreting a pedigree	Interpretation of pedigrees	60	77	0.04
	Concept of obligate carrier	65	75	0.20
	Concept of proband	64	88	0.002
	Modes of inheritance	71	79	0.29
	Concepts of penetrance and expressivity	52	69	0.05
Recognize when to refer a family for cascade genetic testing	Recognize autosomal dominant inheritance	31	56	0.002
	Infer mode of inheritance	28	52	0.003
	Analyse segregation	15	42	0.0002
	Recognize X-linked inheritance	38	56	0.03
	Determine recurrence risk in pedigree	57	88	0.0001
Recognize the appropriate indications to refer a patient for genetic counseling	Clinical predictors of an inherited single gene mutation	12	27	0.02
	Justification for genetic testing	1	29	0.0001
	Roles of a genetic counsellor	36	35	0.94
Interpret the clinical significance of genetic mutations	Recognize clinical consequences: Response to anesthesia	5	23	0.0003
	Recognize syndromic features on physical exam: Marfan syndrome	34	69	0.0001
Recognize how genetic data can change clinical decision-making	Effect of CYP2C19 genotypes on drug metabolism	50	81	0.0002

**Table 3:** Summary of learner feedback by tier (T1-T3) and module (M1-M9) or case number (C1-C10). n, number of respondents; Time, mean time in minutes to complete module; Confidence, confidence to use genetic information in clinical practice; Clarity, perceived clarity of module content; Satisfaction, satisfaction with module content; Likely to practice %, likely to apply genetic knowledge in clinical practice; Likely to consult %, likely to consult with genetic providers.

Module	n	Time(min)	Confidence%	Clarity%	Satisfaction%	Likely to practice %	Likely to consult %
T1 M1	59	21	80	77	88	59	66
T1 M2	27	19	81	77	93	61	71
T1 M3	24	21	76	71	92	63	66
T1 M4	17	21	79	77	100	67	71
T1 M5	16	21	75	70	88	61	75
T1 M6	14	18	86	77	100	67	77
T1 M7	15	20	74	64	93	71	77
T1 M8	14	18	77	66	86	60	66
T1 M9	16	14	77	75	100	67	70
T2 M1	18	19	74	71	100	66	68
T2 M2	14	17	79	80	100	72	75
T2 M3	12	16	67	68	100	66	75
T2 M4	10	18	71	70	90	71	69
T2 M5	9	17	71	84	89	63	65
T3 C1	14	20	78	80	100	67	62
T3 C2	9	21	72	68	100	66	77
T3 C3	10	21	77	79	100	71	75
T3 C4	8	19	81	78	100	71	79
T3 C5	9	18	86	84	89	70	72
T3 C6	9	21	83	81	100	71	78
T3 C7	11	20	79	86	90	68	73
T3 C8	8	19	77	71	88	72	77
T3 C9	10	22	73	79	100	69	75
T3 C10	9	21	80	81	100	73	84
**Means**	**15**	**19**	**72**	**76**	**95**	**67**	**73**

## Data Availability

All primary data from this study will be made available by the authors on reasonable written request.

## Abbreviations

CGC: The UTHealth Adult Cardiovascular Genomics Certificate program
TAD: Thoracic aortic aneurysms and dissections
ISCC-PEG: Inter-Society Coordinating Committee for Practitioner Education in Genomics
TRIG: Training Residents in Genomics
CME: Continuing medical education
CNE: Continuing nursing education
CGTA: CardioGenomic Testing Alliance
ACC: The American College of Cardiology
ELSI: Ethical, Legal, or Social Issues
CPHS: Committee for the Protection of Human Subjects
